# Triple SILAC identified progestin-independent and dependent PRA and PRB interacting partners in breast cancer

**DOI:** 10.1038/s41597-021-00884-0

**Published:** 2021-04-12

**Authors:** Prangwan Pateetin, Gyorgy Hutvagner, Sarah Bajan, Matthew P. Padula, Eileen M. McGowan, Viroj Boonyaratanakornkit

**Affiliations:** 1 Department of Clinical Chemistry, Faculty of Allied Health Sciences, Chulalongkorn University, Bangkok, 10330 Thailand; 2 School of Biomedical Engineering, Faculty of Engineering and Information Technology, University of Technology Sydney, Sydney, Australia; 3 School of Health and Behavioural Sciences, University of the Sunshine Coast, Queensland, Australia; 4 Sunshine Coast Health Institute, Birtinya, Australia; 5 School of Life Sciences and Proteomics Core Facility, Faculty of Science, University of Technology Sydney, Sydney, Australia; 6 School of Life Sciences, University of Technology Sydney, Sydney, Australia; 7 Age-related Inflammation and Degeneration Research Unit, Chulalongkorn University, Bangkok, 10330 Thailand

**Keywords:** Breast cancer, Mass spectrometry, Proteomic analysis

## Abstract

Progesterone receptor (PR) isoforms, PRA and PRB, act in a progesterone-independent and dependent manner to differentially modulate the biology of breast cancer cells. Here we show that the differences in PRA and PRB structure facilitate the binding of common and distinct protein interacting partners affecting the downstream signaling events of each PR-isoform. Tet-inducible HA-tagged PRA or HA-tagged PRB constructs were expressed in T47DC42 (PR/ER negative) breast cancer cells. Affinity purification coupled with stable isotope labeling of amino acids in cell culture (SILAC) mass spectrometry technique was performed to comprehensively study PRA and PRB interacting partners in both unliganded and liganded conditions. To validate our findings, we applied both forward and reverse SILAC conditions to effectively minimize experimental errors. These datasets will be useful in investigating PRA- and PRB-specific molecular mechanisms and as a database for subsequent experiments to identify novel PRA and PRB interacting proteins that differentially mediated different biological functions in breast cancer.

## Abstract (summary)

**Table:** 

Measurement(s)	progesterone receptor • isoform
Technology Type(s)	affinity purification method • stable isotope labeling by amino acids in cell culture
Factor Type(s)	progestin presence • biological replicate

Machine-accessible metadata file describing the reported data: 10.6084/m9.figshare.14179040

## Background & Summary

Estrogen receptor (ER) and progesterone receptor (PR) are used to classify breast cancer histological subtypes and predict hormone therapy responsiveness^1^. The majority of breast cancers express both ER and PR, and the presence of PR is a good prognosis factor in ER-positive breast cancer subtypes^2^. In humans, PR is expressed as two isoforms, PRA and PRB^3^, and their expression ratio is important whereby PR isoforms are functionally distinct and differentially influence tumor phenotypes^4–10^.

**Fig. 1:**
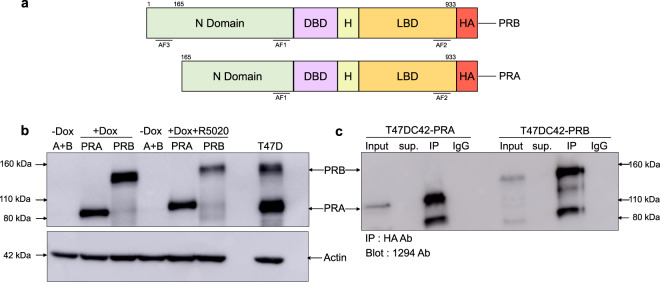
Cell model and Co-IP optimization. (**a**) PRA and PRB protein structure. PRB is a full-length isoform, PRA lacks 164 amino acids at the N-terminus domain. HA-tag was appended at the C-terminus domain of both isoforms. The ligand-binding domain (LBD), hinge region (H), the DNA-binding domain (DBD), and activation function domains (AFs) are indicated. (**b**) Western blot results of PRA and PRB expression in T47DC42-PRA and T47DC42-PRB cells treated (+) or untreated (−) with 1000 ng/mL of Dox or Dox with 10 nM R5020 for 1 h to induce the expression of PRA or PRB proteins. Cell lysates were prepared and immunoblotted with a 1294, PR-specific antibody. Thirty micrograms of protein were loaded in each lane. Ten micrograms of T47D cell lysate was loaded as a positive control. Actin was used as a loading control. (**c**) Co-IP optimization, 1.5 mg of proteins from T47DC42-PRA or T47DC42-PRB treated with 1000 ng/mL of Dox for 24 h were separately IP with HA ab (1:100) and blotted with PR specific 1294 ab. Input, unbound supernatant (sup) and rabbit IgG isotype (IgG) were used as a control.

PRA and PRB isoforms share common N-terminal sequences, DNA binding domain (DBD), ligand-binding domain (LBD), and two activation functional domains, AF1 and AF2 (Fig. 1)^11^. However, PRB contains an extra 164 amino acids at the N-terminal encompassing an extra AF3. PRB-AF3 domain contributes to strong transcriptional activity by suppressing the activity of an inhibitory domain (ID) within the common PRA/PRB N-terminal^12^. Both isoforms function through nuclear and extra-nuclear signaling, with extra-nuclear signaling mostly mediated by PRB^13^. PR serves as a DNA binding partner modifying ER transcriptional activity, whereby PRA inhibits ER chromatin binding while PRB helps redistributes ER chromatin binding^14^.

Numerous studies have attempted to identify PR interactome in breast cancer using two-hybrid interaction techniques or affinity capture-westerns^15–17^. A study using RIME coupled with label-free mass spectrometry identified PR isoforms binding to DNA and coregulators complexes focused on ligand-dependent interacting partners^7^. However, liganded- and unliganded-PRA and -PRB can differentially regulate distinct and overlapping gene expression in breast cancer^18,19^. Our previous studies showed unliganded-PR isoforms differentially regulate breast cancer proteomes; unliganded-PRA regulates proteins involved in the TCA cycle while unliganded-PRB regulates proteins involved in cell cycle and apoptosis^20^.

Here we identified PRA and PRB interacting proteins in the presence and absence of progestin using sensitive, reliable technology. Tet-inducible PRA and PRB constructs were expressed in PR-null T47DC42 breast cancer cells using lentiviral transduction (see Materials and Methods). To specifically purify PRA and PRB complex proteins, a HA tag (YPYDVPDYA) was attached to the PR-isoform C-terminus (Fig. 1a). Similar levels of PRA and PRB were induced with doxycycline (Dox). Treatment with synthetic progesterone (R5020) decreased PRA and PRB levels (Fig. 1b). Co-immunoprecipitation (Co-IP) of PRA and PRB complexes were successfully achieved using the HA-tag-specific monoclonal antibody (Fig. 1c). The tet-inducible PR-isoform models were previously characterized showing normal transcription, localization, and function^20,21^.

We applied Stable Isotope Labeling with Amino acids in Cell culture (SILAC) coupled with coimmunoprecipitation (Co-IP)^22^. SILAC with high-affinity purification provided a highly effective method to identify protein-protein interactions with lower nonspecific binding than other traditional affinity purifications.

**Fig. 2:**
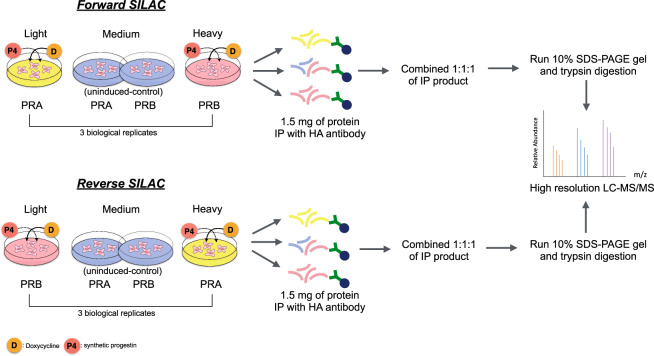
Experimental workflow. Summary of the experimental work-flow applied to investigate PRA and PRB interacting partners, both ligand-independent (Dox treatment) and ligand-dependent (Dox with R5020 treatment). Forward SILAC, PRA: Light, PRB: Heavy. Reverse SILAC, PRA: Heavy, PRB: Light. Equal amounts of uninduced-PRA and uninduced-PRB labeled in medium were combined and used as a background for each experiment. Equal amounts of protein lysate from Light PRA and Heavy PRB and Medium control cells were immunoprecipitated with HA-tag antibody-conjugated with agarose beads. Repeated washings were performed to remove nonspecific protein bindings. PR and PR-interacting protein complexes were eluted using laemmli buffer. Eluted proteins from light, medium, and heavy were mixed 1:1:1 and separated by SDS-polyacrylamide gel electrophoresis (SDS-PAGE). Gels were stained with Coomassie blue stain, cut into slices, and digested with trypsin before injecting into high-resolution LC-MS/MS analysis.

To identify a list of interacting proteins with high confidence, we conducted three-forward and three-reverse SILAC experiments in the presence and absence of progestin to minimize experimental bias and errors: forward SILAC, PRA was labeled with Light isotope, PRB was labeled with Heavy isotope; reverse SILAC experiments, PRA and PRB labeling were swapped. We combined the equivalent amount of protein from uninduced-PRA and uninduced-PRB cells cultured in intermediate SILAC medium as controls to help minimize nonspecific protein bindings. The experimental workflow is described, Fig. 2.

**Fig. 3:**
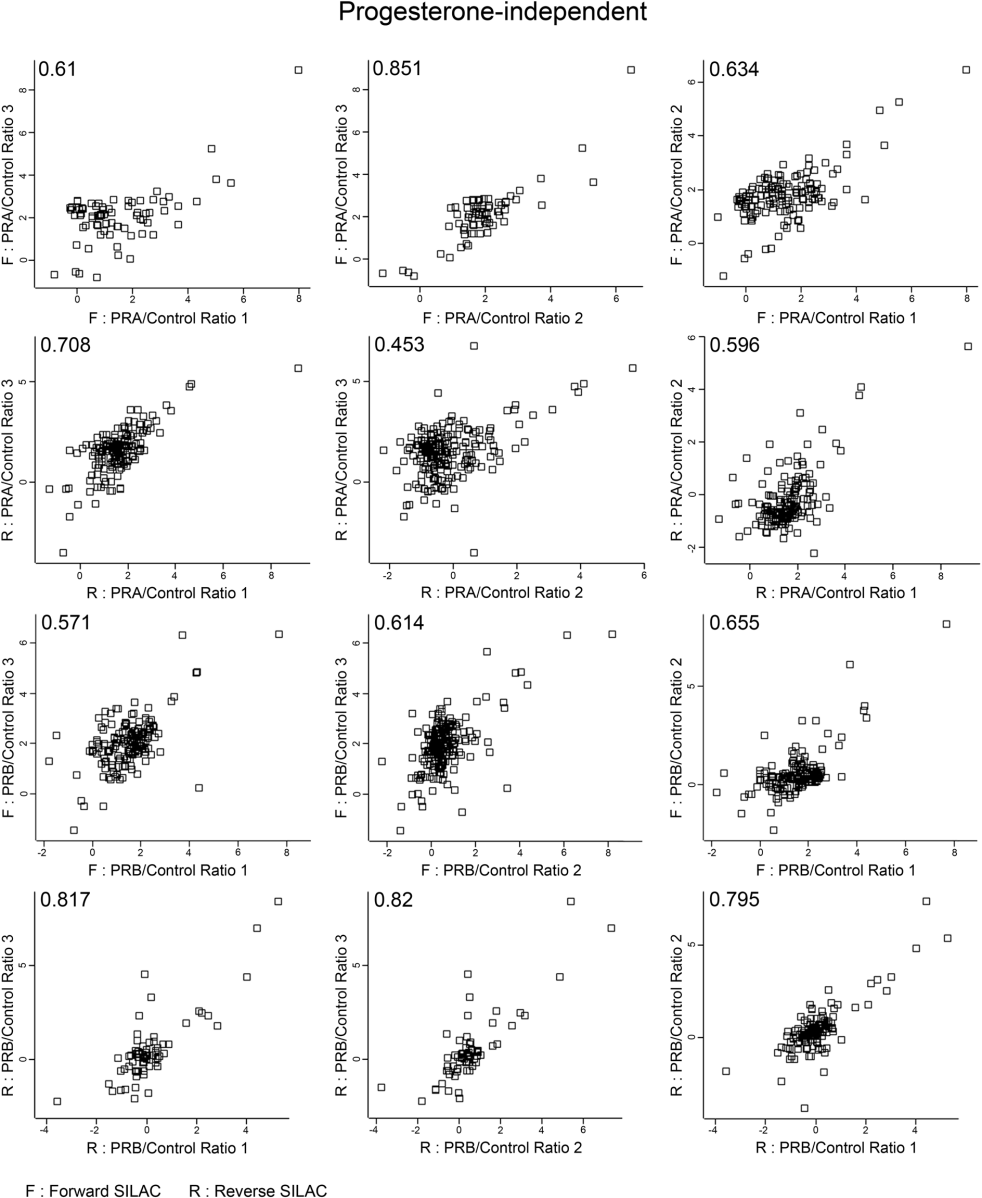
Scatter plots of log2 SILAC ratio comparing across replicates in progesterone-independent condition. Scatter plot shows correlation coefficients between log2 SILAC ratio of progesterone-independent across replicates both forward (F) and reverse (R) SILAC conditions. Values indicate Pearson correlation coefficients.

**Fig. 4:**
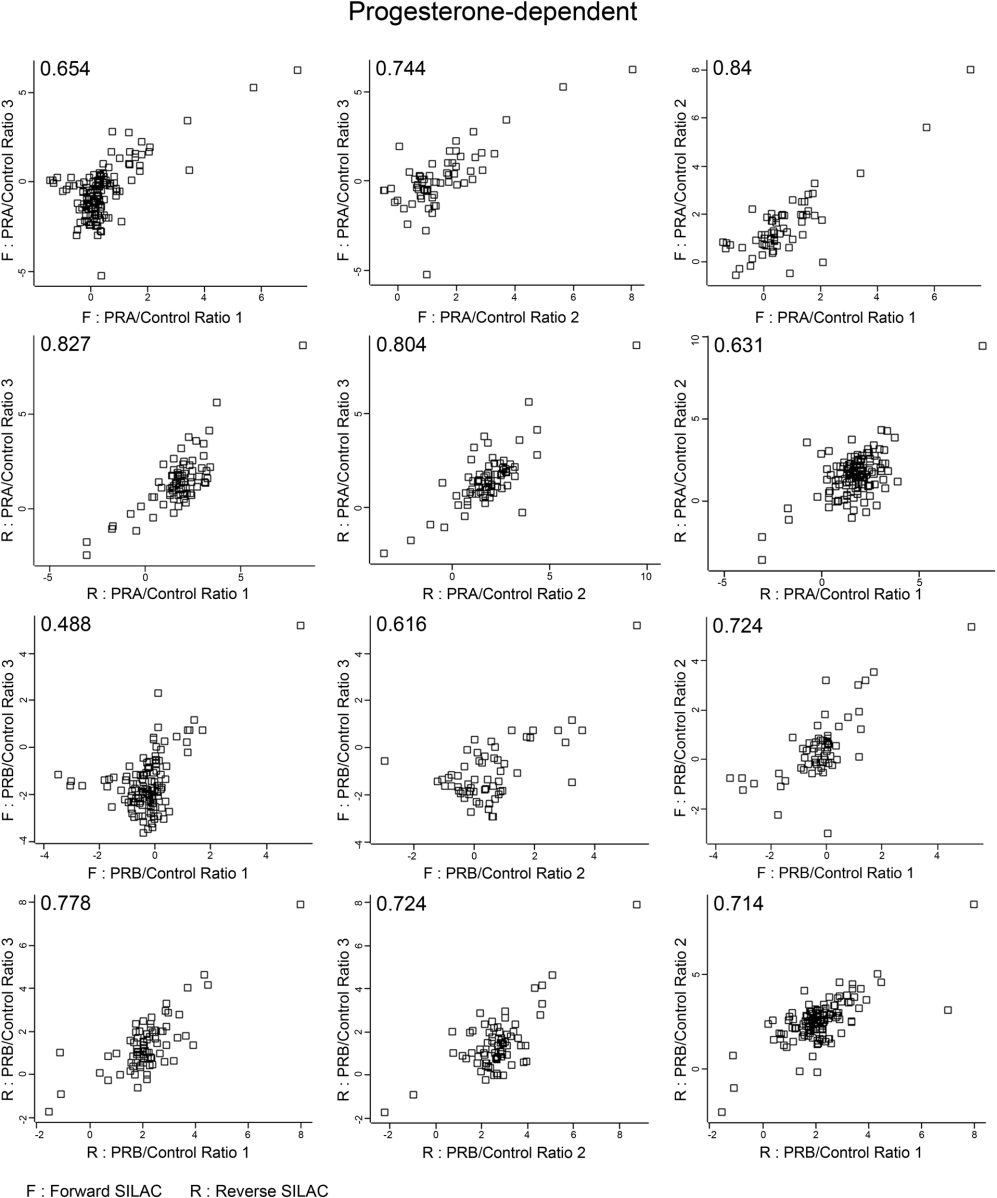
Scatter plots of log2 SILAC ratio comparing across replicates in progesterone-dependent condition. Scatter plot shows correlation coefficients between log2 SILAC ratio of progesterone-dependent across replicates both forward (F) and reverse (R) SILAC conditions. Values indicate Pearson correlation coefficients.

**Fig. 5:**
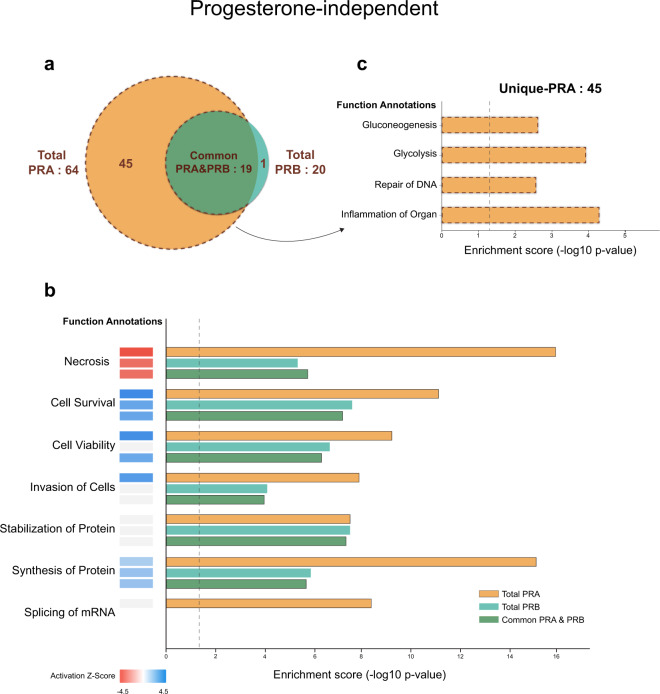
Functional enrichment analysis of progesterone-independent PRA and PRB interacting partners by IPA. (**a**) Venn diagram indicating the number of PRA and PRB interacting proteins both total, common, and unique interactors of PRA and PRB. (**b**) Total candidate interacting partners of PRA&PRB, common PRA&PRB, and (**c**) only unique-PRA interacting partners were used to predict the functional enrichment analysis. The vertical axis (y-axis) corresponds to the functional annotations. The horizontal axis (x-axis) corresponds to the enrichment score (-log10 p-value) based on the Fisher’s exact test (p-value < 0.05), the dash line indicates the minimum significance level (p-value < 0.05). Activation state (z-score) was indicated in color scale between −4.5 to 4.5, red color indicates negative z-score and blue color indicates positive z-score.

**Fig. 6:**
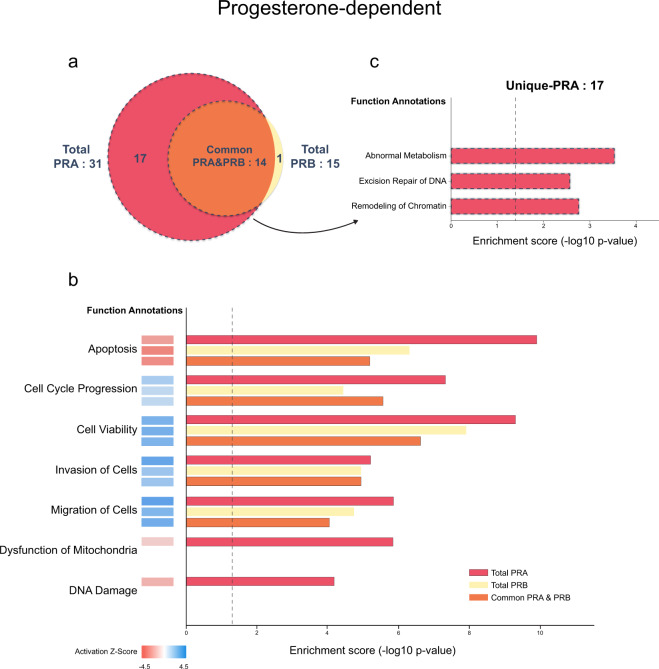
Functional enrichment analysis of progesterone-dependent PRA and PRB interacting partners by IPA. (**a**) Venn diagram indicating the number of PRA and PRB interacting proteins both total, common, and unique interactors of PRA and PRB. (**b**) Total candidate interacting partners of PRA&PRB, common PRA&PRB, and (**c**) only unique-PRA interacting partners were used to predict the functional enrichment analysis. The vertical axis (y-axis) corresponds to the functional annotations. The horizontal axis (x-axis) corresponds to the enrichment score (-log10 p-value) based on the Fisher’s exact test (p-value < 0.05), the dash line indicates the minimum significance level (p-value < 0.05). Activation state (z-score) was indicated in color scale between −4.5 to 4.5, red color indicates negative z-score and blue color indicates positive z-score.

We evaluated the correlation across replicates using Pearson’s correlation and found the average correlation of progesterone-independent and dependent at 0.677 and 0.712, respectively, as shown in Figs. 3, 4. Analysis by LC-MS/MS in progestin-independent and dependent conditions identified a total of 742 proteins and 646 proteins, respectively. In the absence of progestin, we identified 210 and 202 interacting PRA and PRB interacting partners that were progestin-independent. In the presence of progestin, we identified 141 and 135 PRA and PRB interacting partners that were progestin-dependent. To identify high confidence of PRA and PRB interacting partners, only PRA and PRB interacting partners detected in at least 4 out of 6 replicates were allowed for statistical analysis with a one-sample t-test (p-value < 0.05). Protein with p-value < 0.05 and showed a minimum fold-change of greater than 2 (log2 SILAC ratio ≥ 1) were allowed in significant candidate protein lists, which were provided as described in the data record. We found 64 and 20 of PRA and PRB, respectively, significant interacting partners that were progestin-independent and found 31 and 15 of PRA and PRB, respectively, significant interacting partners that were progestin-dependent. We identified known interacting partners of PRA and PRB including HSP90, HSP70, DDX5, FKBP5, and PARP1 proteins, and others as listed^23,24^. We also identified several novel PRA and PRB interacting partners under ligand-independent and dependent conditions. Since we applied stringent criteria to rule out nonspecific binding and performed traditional immunoprecipitation without cross-linking agents, interacting partners identified in this study are likely PRA or PRB binding proteins with high-affinity stable protein interactors. According to the list, we found more PRA interacting partners in the absence of ligand, consistent with a previous study that found PRA is a more active isoform compared to PRB under progestin-independent condition^19^. Moreover, these two receptors exhibited distinct conformations and PRA contain an inhibitory domain (ID), prompting PRA to function as a strong ligand-dependent transdominant repressor of steroid hormone receptor transcriptional activity^25^. Since progestin-bound receptors get phosphorylated and degraded via a proteasome-dependent pathway and PRB rapidly degrades as compared to PRA (Fig. 1b)^26,27^, we found fewer progestin-dependent PRB interacting partners compared to that of PRA. The majority of PRB interacting partners 19 out of 20 proteins (95%) and 14 out of 15 (93%) proteins of progesterone-independent and dependent, respectively, are a subset of PRA progesterone-independent and dependent interacting partners as shown in the Venn diagrams in Figs. 5a, 6a. Together, our data support the small number of potential PRB interactors as compared to those of PRA.

Ingenuity Pathway Analysis (IPA) showed that PRA and PRB interacting proteins enriched similar pathways but differences in significance value, except for the splicing of mRNA pathway that was only involved in unliganded-PRA, Fig. 5b. Moreover, unique interactor proteins of unliganded-PRA are involved in gluconeogenesis, glycolysis, among others, as shown in Fig. 5c. In the presence of progestin, proteins preferentially interacting with progesterone-bound PRA and PRB enriched similar pathways but showed differences in significance value, except for proteins in DNA damage and dysfunction of mitochondria pathways that were only interacting in progesterone-bound PRA, Fig. 6b. Moreover, unique interacting proteins of progesterone-bound PRA are involved in abnormal metabolism, excision repair of DNA, remodeling of chromatin as shown in Fig. 6c.

Importantly, we discovered novel PRA and PRB interacting partners in progesterone-independent and dependent conditions. Our dataset of PRA and PRB interacting partners will be useful in investigating the molecular mechanisms of PRA and PRB in breast cancer. These new PRA and PRB interactome data will serve as molecular resources benefiting future interrogation into the PRA and PRB mediate breast cancer progression.

## Methods

### Inducible HA-tag PRA and HA-tag PRB with a Tet-on lentiviral system

To identify PRA and PRB interacting partners, we applied a Tet-on lentiviral transduction technique to transduce PRA or PRB into T47DC42 (ER -, PR-) breast cancer cells, as previously described^20,28^. To check the expression of PRA and PRB, T47DC42-PRA and T47DC42-PRB 200,000 cells were plated in phenol red-free DMEM (Dulbecco’s modified eagle medium), 5% DCC-FBS (Dextran-coated charcoal-stripped FBS; Gibco/Life Technologies) and 1% penicillin/streptomycin (PenStrep) in a 6-well plate and incubated overnight. The next day, cells were treated with 1000 ng/mL of Dox for 24 h or Dox with 10 nm R5020 for 1 h. Cells were washed once with ice-cold PBS and lysed with RIPA lysis buffer (Merck Millipore) containing proteinase inhibitor cocktail (Roche). Cells were scraped and the lysate was collected and rotated end-over-end for 30 min at 4 °C. The supernatant was collected, and protein concentration was performed using a Bradford assay (Bio-Rad). Similar amounts of protein were separated on a 10%SDS-PAGE gel, and proteins were transferred onto PVDF membranes. Blots were probed with (1:2500 v/v) 1294 PR, a mouse-specific monoclonal primary antibody recognizing PRA and PRB^29^, Actin antibody (1:10,000 v/v, Santa Cruz Biotechnology), Protein bands were visualized on Amersham AI600 imager (GE Healthcare) via chemiluminescence reaction using Pierce® ECL Immunoblotting Substrate (Thermo Scientific).

### Co-immunoprecipitation coupled triple SILAC identifies PRA and PRB interacting partners

SILAC culture medium was prepared as followed, SILAC DMEM (Thermo Scientific) supplemented with 5% dialyzed fetal calf serum (Invitrogen), 10 ug/mL of insulin (Gibco/Life Technologies), and 1% PenStrep. ‘Light’ labeling contained normal isotopic abundance lysine (146 mg/L) and arginine (84 mg/L). ‘Medium’ labeling contained ^2^H_4_-lysine (150 mg/L) and ^13^C_6_-arginine (86 mg/L), and ‘Heavy’ labeling contained ^13^C_6_^15^N_2_-lysine (152 mg/L) and ^13^C_6_^15^N_4_-arginine (88 mg/L). All amino acids were purchased from Silantes GmbH. Cells were cultured in SILAC medium for at least 5 passages to ensure the full incorporation of SILAC amino acids into the proteins (greater than 95% incorporation). Labeling incorporation was verified by mass spectrometry. In forward SILAC; T47DC42-PRA and T47DC42-PRB were cultured in ‘Light’ and ‘Heavy’ SILAC labeling cell culture media. Uninduced-PRA and PRB (no Dox treatment) were grown in ‘Medium’ SILAC labeled cell culture media as a control. PRA and PRB labeling were swapped in the reverse SILAC experiments except for the control. To investigated PRA and PRB interacting partners in ligand-independent experiments, cells expressing either PRA or PRB were seeded at 500,000 cells/well in 100 mm cell culture dish and incubated for 24 h to achieve 70–80% confluency. Cells were treated with 1000 ng/mL of Dox for 24 h. For ligand-dependent experiments, cells were pretreated with 1000 ng/mL of Dox for 24 h and treated with 10 nM of R5020 for an additional 1 h. Ethanol was used as the vehicle control for all experiments. On harvesting, cells were washed one time in cold PBS and then scraped in cold IP lysis buffer [1% (vol/vol) Nonidet P-40, 30 mM KCl, 50 mM Tris-HCl (pH 7.4)], 1 mM Na_3_VO_4_, and with 1X protease inhibitors (Roches) and total cell lysate were rotated end-over-end at 4 °C for 30 min. Cell lysate was centrifuged at 13000 rpm at 4 °C for 10 min and collected the supernatant. Protein concentration was analyzed by Pierce BCA protein assay kit (Thermo Scientific) following the manufacturer’s instructions. 1.5 milligram of protein from each Light, Medium, and Heavy cell culture media were separately immunoprecipitated using the HA-tag antibody (Cell Signaling Technology, dilution 1:100) at 4 °C overnight with end-over-end rotation. 50 uL of Dynabeads Protein-G (Invitrogen/Life Technologies) were washed three times with PBS and resuspended in IP lysis buffer. To precipitate HA-tag protein complexes, dynabeads were added and rotated at 4 °C for 1.5 h. Bead and protein complexes were washed three times with 500 uL of wash buffer [1% (vol/vol) Nonidet P-40, 150 mM NaCl, 50 mM Tris (pH 7.4)]. The beads were suspended in 30 uL of 2XSDS loading buffer with 10% DTT and denatured at 95 °C for 10 min. To collect Co-IP protein complexes, beads were centrifuged at 13000 rpm for 10 minutes.

The Co-IP products from light, medium, and heavy were then combined 1:1:1 and short-run on 10% SDS-PAGE gels for 10 min. Each gel was fixed with 50% methanol–7% acetic acid for 1 h with gentle shaking before staining with Coomassie blue for 1 h and destained with milliQ water overnight. In gel digestion was performed as previously described^30^. Briefly, individual gels were cut into 1 × 1 mm pieces, then 50% acetonitrile (ACN)–50 mM NH_4_HCO_3_ was added and incubated for 10 min at room temperature to de-stained the color. Gel pieces were reduced and alkylated with 5 mM tributylphosphine and 20 mM acrylamide (Sigma) in 100 mM NH_4_HCO_3_ for 90 min at room temperature. Then the gel pieces were dehydrated with 100% ACN before proteins were digested with trypsin (Sigma Proteomics grade) at 37 °C overnight. After trypsin digestion, the supernatant containing the peptides were sonicated in a water-bath for 10 min before collection. The sonication step was repeated after adding 100 µL of 50% ACN–0.1% formic acid and peptides solution was collected and combined with the previous one. The peptide volume was reduced to 30 µL by rotary evaporation and centrifuged at 14,000 g for 10 min to remove interfering materials before LC-MS/MS analysis.

### Nano LC-MS/MS

The nano LC-MS/MS was set up as previously described^31^. The Acquity M-class nanoLC system (Waters, USA) was used to analyze the peptide sample. A 5 µL aliquot of the sample was loaded onto a nanoEase Symmetry C18 trapping column (180 µm × 20 mm) over a 3 minute period at 15 µL/min. The sample was then washed onto a PicoFrit column (75 µmID × 300 mm; New Objective, Woburn, MA) which was packed with Magic C18AQ resin (3 µm, Michrom Bioresources, Auburn, CA). The eluted peptides were loaded into the mass spectrometer (Q Exactive Plus mass spectrometer; Thermo Scientific). The program configuration was: 5–30% MS buffer B (98% Acetonitrile + 0.2% Formic Acid) for a time period of 90 minutes, then 30–80% MS buffer B for 3 minutes, followed by 80% MS buffer B over 2 minutes, and then 80-5% for a further 3 min. The peptides obtained after elution were ionised at 2400 V. The Data Dependant MS/MS (dd-MS^2^) investigation was executed using a survey scan of 350–1500 Da performed at 70,000 resolution for peptides of charge state 2 + or higher with an AGC target of 3e6 and maximum injection time of 50 ms. Using an isolation window of 1.4 m/z, an AGC target of 1e5 and a maximum injection time of 100 ms, the top 12 peptides were chosen and fragmented in the HCD cell. The selected fragments were scanned using the Orbitrap analyzer at a resolution of 17,500. The resulting product ion fragment masses were measured (mass range of 120–2000 Da). The precursor peptide mass was subsequently excluded for 30 seconds.

### Data analysis

A total of 12 raw files corresponding to three forward and three reverse SILAC of both progestin independent and dependent (Table 1) were analyzed using the MaxQuant software suite 1.6.0.16 (www.maxquant.org)^32^. We processed six replicates with both forward and reverse labeled samples together and searched against an in silico tryptic digest of human proteins from the UniProt sequence database (September 1, 2017) by the Andromeda search engine. Enzyme specificity was set to trypsin with only tryptic peptides with a minimum of seven amino acids in length and a maximum of two missed cleavages considered. A precursor mass tolerance of 20 ppm and a fragment mass tolerance of 0.5 Da with an FDR < 0.01 at the level of proteins, peptides, and modifications were set for mass spectra searching. The search included propionamide (C) as a fixed modification, acetylation of protein amino (N)-termini, oxidation of methionine, deamidation of asparagine and glutamine, medium (Arg + 6, Lys + 4), and heavy (Arg + 10, Lys + 8) isotope labeling were set as variable modification. The “proteinGroups.txt” file produced by MaxQuant was further analyzed in Perseus (version 1.6.1.1). The SILAC ratios were log2-transformed and proteins from the reverse database, proteins only identified by site, and contaminants were removed. Only proteins identified in at least four of the six replicates were allowed for further analysis. Statistical analysis was performed by employing a one-sample t-test (p-value < 0.05), when log2 SILAC ratios (L/M and H/M) were compared against a value of 0 (control, log2(1)). Moreover, proteins with an average SILAC ratio ≥ 1 were only considered as high confidence protein partners. The use of SILAC ratio cut-off plus the p-value < 0.05 as criteria instead of applying multiple testing correction could help reduce the false positive without excluding true-positive interacting partners in quantitative proteomics^33^.

**Table 1:** Summary of the protocols and proteomics dataset of PRA and PRB both progesterone-independent and dependent.

Treatment	Protocol 1	Protocol 2	Protocol 3	Data
P4 independent replicate 1	Forward SILAC PRA: L PRB: H Control: M	Co-Immunoprecipitation	Nano LC-MS/MS	PXD023920
P4 independent replicate 2	Forward SILAC PRA: L PRB: H Control: M	Co-Immunoprecipitation	Nano LC-MS/MS	PXD023920
P4 independent replicate 3	Forward SILAC PRA: L PRB: H Control: M	Co-Immunoprecipitation	Nano LC-MS/MS	PXD023920
P4 independent replicate 1	Reverse SILAC PRA: H PRB: L Control: M	Co-Immunoprecipitation	Nano LC-MS/MS	PXD023920
P4 independent replicate 2	Reverse SILAC PRA: H PRB: L Control: M	Co-Immunoprecipitation	Nano LC-MS/MS	PXD023920
P4 independent replicate 3	Reverse SILAC PRA: H PRB: L Control: M	Co-Immunoprecipitation	Nano LC-MS/MS	PXD023920
P4 dependent replicate 1	Forward SILAC PRA: L PRB: H Control: M	Co-Immunoprecipitation	Nano LC-MS/MS	PXD023920
P4 dependent replicate 2	Forward SILAC PRA: L PRB: H Control: M	Co-Immunoprecipitation	Nano LC-MS/MS	PXD023920
P4 dependent replicate 3	Forward SILAC PRA: L PRB: H Control: M	Co-Immunoprecipitation	Nano LC-MS/MS	PXD023920
P4 dependent replicate 1	Reverse SILAC PRA: H PRB: L Control: M	Co-Immunoprecipitation	Nano LC-MS/MS	PXD023920
P4 dependent replicate 2	Reverse SILAC PRA: H PRB: L Control: M	Co-Immunoprecipitation	Nano LC-MS/MS	PXD023920
P4 dependent replicate 3	Reverse SILAC PRA: H PRB: L Control: M	Co-Immunoprecipitation	Nano LC-MS/MS	PXD023920

L: Light labeling, M: Medium labeling, and H: Heavy labeling.

Biological functions associated with PRA and PRB interacting partners were projected using IPA software (Qiagen Inc., USA, https://www.qiagenbioinformatics.com/products/ingenuity-pathway-analysis)^34^. Fisher’s exact test was performed to calculate p-value, and a p-value < 0.05 was considered statistically significant.

## Data Records

The mass spectrometry proteomics data have been deposited to the ProteomeXchange Consortium via the PRIDE^35^ partner repository with the dataset identifier PXD023920^36^. The dataset includes 12 raw files, 2 MaxQuant parameter files (mqpar.xml), and 2 result files, “proteinGroups.txt” of progesterone-independent and dependent conditions. 12 raw files represent 3 biological replicates of forward and 3 biological replicates of reverse SILAC from progesterone-dependent and independent conditions (Table 1). Raw files are non-processed outputs from Q-Exactive plus mass spectrometer. The short-run of gel images and interacting candidate proteins with statistical significance of PRA and PRB both progesterone-independent and dependent were provided via the figshare repository^37^. The protein interactions data have been submitted to the IMEx (http://www.imexconsortium.org) consortium through IntAct and assigned the identifier IM-28705^38^.

## Technical Validation

Cell lines used in our study were free from mycoplasma contamination and were routinely tested for mycoplasma contamination using the MyoAlert^TM^ mycoplasma detection kit (Lonza, Switzerland). Our cell model for PRA and PRB interacting partners was characterized in our previous study^20^. In brief, we successfully induced similar amounts of PRA and PRB as verified by Western blots (Fig. 1b). The biological function of inducible PRA and PRB were characterized and were shown to function similar to PRA and PRB expressed in PR-positive breast cancer cells. Co-IP technique using anti-HA-tag was successfully optimized, as showed in Fig. 1c.

T47DC42, PR-null breast cancer cells were derived from ER/PR-positive T47D cells through long term culture in estrogen deprived medium, resulting in a T47D variant with low to no ER/PR expression^39^. T47D was suggested as an ideal breast cancer cells model to study progesterone signaling as it reflects a luminal A-ER and PR positive subtype, which is the most common type of breast cancer^40,41^. Thus, T47DC42 cells–T47D subclone are suitable for re-expressed PR isoforms as they should contain appropriate factors required for PR function or response to progestin. Moreover, using the Tet-inducible PR expression system, we can induce and identify individual PRA and PRB interacting partners under both progestin-independent and dependent conditions. Since we individually re-expressed PRA or PRB isoforms in PR-null breast cancer cells, our potential limitation is that only homodimer not heterodimer of PRA and PRB interacting partners are investigated.

We applied triple SILAC labeling, Light, Medium, and Heavy, to distinguish the protein interacting partners between PRA and PRB and also nonspecific binding by comparing the ratio measurement of Light/Medium or Heavy/Medium. To ensure the reliability of our data, we applied both three biological replicates forward, and three biological replicates reverse of each condition of SILAC to help enhance the reliability and reproducibility and also correct the experimental errors by averaging ratios measurement of identifying interacting partners. Moreover, we applied the triple SILAC labeling and used the Medium labeling—uninduced-PRA combined with uninduced-PRB as controls for the experiments to reduce the nonspecific binding results that often occur during the traditional IP. To reduce false identification of PR interacting proteins, only proteins that met the following criterion; detected in at least 4 out of 6 replicates, showed the statistical significance of SILAC ratio p-value < 0.05, with a minimum fold-change > 2 (log2 SILAC ratio ≥ 1) were considered as highly confident PR interacting partners. The average SILAC ratio cut-off was based on Co-IP validation of an unliganded-PRA interacting partner, splicing factor proline and glutamine-rich (SFPQ), which showed the lowest average SILAC ratio 1.00 (data not shown). Moreover, correlation coefficients of log2 SILAC ratio across replicates indicated reliability and reproducibility between replicates as the average correlation of progesterone-independent and dependent are 0.677 (value range 0.453–0.851) and 0.712 (value range 0.488–0.84), respectively shown in the scatter plot, Figs. 3, 4. Finally, the IPA functional annotations of PRA interacting partners – glycolysis, abnormal metabolism, and remodeling of chromatin, similar to previous studies that identified PRA-rich breast cancer cells expressed proteins that involved in cell metabolism and chromatin remodeling processes^20^. Moreover, in a proteomic analysis of the mouse hypothalamus-PRA interacting partners showed enrichment of proteins involved cell metabolism^42^.

## Data Availability

The presented data were analyzed with the following software and applications: 1.  All files were analyzed using the MaxQuant software suite 1.6.0.16 (www.maxquant.org). 2.  Quantitative data analysis and Pearson correlation were performed with Perseus (www.perseus-framework.org). 3.  Functional analysis was performed with the IPA software (Qiagen Inc.,USA, https://www.qiagenbioinformatics.com/products/ingenuity-pathway-analysis).
