# Author Correction: Triple SILAC identified progestin-independent and dependent PRA and PRB interacting partners in breast cancer

**DOI:** 10.1038/s41597-021-00928-5

**Published:** 2021-05-21

**Authors:** Prangwan Pateetin, Gyorgy Hutvagner, Sarah Bajan, Matthew P. Padula, Eileen M. McGowan, Viroj Boonyaratanakornkit

**Affiliations:** 1 Department of Clinical Chemistry, Faculty of Allied Health Sciences, Chulalongkorn University, Bangkok, 10330 Thailand; 2 School of Biomedical Engineering, Faculty of Engineering and Information Technology, University of Technology Sydney, Sydney, Australia; 3 School of Health and Behavioural Sciences, University of the Sunshine Coast, Queensland, Australia; 4 Sunshine Coast Health Institute, Birtinya, Australia; 5 School of Life Sciences and Proteomics Core Facility, Faculty of Science, University of Technology Sydney, Sydney, Australia; 6 School of Life Sciences, University of Technology Sydney, Sydney, Australia; 7 Age-related Inflammation and Degeneration Research Unit, Chulalongkorn University, Bangkok, 10330 Thailand

**Keywords:** Breast cancer, Mass spectrometry, Proteomic analysis

Correction to: *Scientific Data* 10.1038/s41597-021-00884-0, published online 12 April 2021

The original version of this Data Descriptor omitted the following author from the Author List: Sarah Bajan. This error has now been corrected in both the PDF and HTML versions of the Article.

